# A mobile phone based alarm system for supervising vital parameters in free moving rats

**DOI:** 10.1186/1756-0500-5-119

**Published:** 2012-02-23

**Authors:** Kristine Kellermann, Matthias Kreuzer, Adem Omerovich, Franziska Hoetzinger, Eberhard F Kochs, Bettina Jungwirth

**Affiliations:** 1 Klinikum rechts der Isar, Klinik für Anaesthesiologie, Ismaninger Str. 22, 81675 Munich, Germany

**Keywords:** Telemetry, Rat, Surveillance, Refinement, Reduction

## Abstract

**Background:**

Study protocols involving experimental animals often require the monitoring of different parameters not only in anesthetized, but also in free moving animals. Most animal research involves small rodents, in which continuously monitoring parameters such as temperature and heart rate is very stressful for the awake animals or simply not possible. Aim of the underlying study was to monitor heart rate, temperature and activity and to assess inflammation in the heart, lungs, liver and kidney in the early postoperative phase after experimental cardiopulmonary bypass involving 45 min of deep hypothermic circulatory arrest in rats. Besides continuous monitoring of heart rate, temperature and behavioural activity, the main focus was on avoiding uncontrolled death of an animal in the early postoperative phase in order to harvest relevant organs before autolysis would render them unsuitable for the assessment of inflammation.

**Findings:**

We therefore set up a telemetry-based system (Data Science International, DSI™) that continuously monitored the rat's temperature, heart rate and activity in their cages. The data collection using telemetry was combined with an analysis software (Microsoft excel™), a webmail application (GMX) and a text message-service. Whenever an animal's heart rate dropped below the pre-defined threshold of 150 beats per minute (bpm), a notification in the form of a text message was automatically sent to the experimenter's mobile phone. With a positive predictive value of 93.1% and a negative predictive value of 90.5%, the designed surveillance and alarm system proved a reliable and inexpensive tool to avoid uncontrolled death in order to minimize suffering and harvest relevant organs before autolysis would set in.

**Conclusions:**

This combination of a telemetry-based system and software tools provided us with a reliable notification system of imminent death. The system's high positive predictive value helped to avoid uncontrolled death and facilitated timely organ harvesting. Additionally we were able to markedly reduce the drop out rate of experimental animals, and therefore the total number of animals used in our study. This system can be easily adapted to different study designs and prove a helpful tool to relieve stress and more importantly help to reduce animal numbers.

## Findings

Study protocols involving laboratory animals often require behaviour to be monitored unrestricted before and after experimental procedures. Especially postoperative monitoring is often complicated by movement artifacts and handling distress considerations.

We conducted a study that aimed at continuously monitoring physiologic parameters in rats in the early postoperative phase after experimental cardiopulmonary bypass (CPB) with 45 min of deep hypothermic circulatory arrest (DHCA). The second end point aimed at investigating the inflammatory reaction to this particular kind of global ischemia in the heart, lungs, liver and kidney. In order to obtain the relevant organs in the situation of an impending death after CPB with DHCA, we sought to develop a system by which we could safely and reliably monitor vital signs of free moving rats after CPB with DHCA. Aim was to minimize suffering of the animal via euthanasia when necessary, simultaneously enabling us to harvest the organs in time for assessment of inflammation, before autolysis begins.

To monitor animals without excessive handling them, devices such as implantable transmitters and telemetry become necessary. By combining continuous data acquisition with real time data analysis, Microsoft Outlook™, and a webmail program (GMX) with a text message-notification service, a simple telemetry device application can be used as a physiologic parameter monitoring surveillance tool. This arrangement allows definition of individual alarm thresholds according to parameter-specific limits, it creates a continuous stream of data, and minimizes handling stress. Whenever a parameter crosses the alarm threshold, the experimenter is instantaneously notified via an automatically generated text message sent to his mobile phone so that he is able to tend to the respective animal.

After approval by the Regierung von Oberbayern (reference number 55.2-1-54-2532-20-10, according to EU directives 86/609/EEC and 2010/63/EU), 92 female Sprague Dawley rats were subjected to experimental cardiopulmonary bypass with deep hypothermic circulatory arrest. Surgery was carried out as previously described [1].

Briefly, rats were intubated and ventilated with oxygen in air FiO_2 _= 40% (PaCO_2 _at 32 - 40 mmHg). Surgical sites were infiltrated with 2% xylocaine. During surgical preparation anaesthesia was maintained with 2.5% isoflurane and 5 μg fentanyl boli. Telemetry transmitters were implanted intraperitoneally, the two ECG leads externalized through the abdominal muscles according to the manufacturer's surgical manual. The negative lead was placed and anchored with non-absorbable sutures (Prolene 7.0, Ethicon, Germany) over the right pectoral muscle. The positive lead was placed and anchored over the left caudal rib region. After placement of the biopotential leads, the abdominal wall was closed (Vicryl, 3-0, Ethicon, Germany) with the transmitter's suture rib incorporated into the closure. After closing the skin incisions, animals were allowed to recover from anaesthesia. When animals resumed spontaneous breathing, the tracheas were extubated and the rats returned to their cages within one hour to begin monitoring.

### Telemetry

The system consists of the telemetry transmitter CTA-F40^®^, that monitors ECG, temperature and activity (DSI, St Paul, USA), the Dataquest^® ^PCI card (DSI, St Paul, USA), connected to the data exchange matrix. Data exchange matrices automatically detect and forward model and serial number of the respective receiver (DSI PhysioTel^® ^RPC-1, DSI, St Paul, USA) used to collect telemetry information fed into the corresponding software (Dataquest^® ^A.R.T. 4.0, DSI, St Paul, USA). With this set up, eight receivers can be integrated into the same alert system, enabling continuous monitoring of eight animals simultaneously. All receivers were connected to this matrix. To each animal that is returned to the animal room, a transmitter and a receiver need to be associated. First, before data acquisition starts, the transmitter has to be configured for the species used and associated with a unique animal ID. Then the according receiver has to be configured for the transmitter type and its serial number.

### Data acquisition and alarm routine

The Dataquest^® ^A.R.T 4.0 software records vital and activity parameters from all implanted rats in sequential order. A Microsoft Excel 2003 macro (Alarm.xls; Microsoft, Redmond, WA, USA) was designed to continuously supervise the trend data from specific parameters such as heart rate for each animal from Dataquest. Trend data are available every 60 s. The acquired values are compared versus defined parameter thresholds, in our case a heart rate of 150 bpm. If the heart rate drops below this threshold value, an alarm email is automatically released to the defined webmail account (in this case a GMX address), from which an instant message is sent to the experimenter. This occurs within two minutes of the inciting event. Since the initially set threshold of a heart rate of zero did not reliably trigger alarms due to measurement artefacts, it was eventually revised to a threshold of 150 bpm.

**Figure 1:**
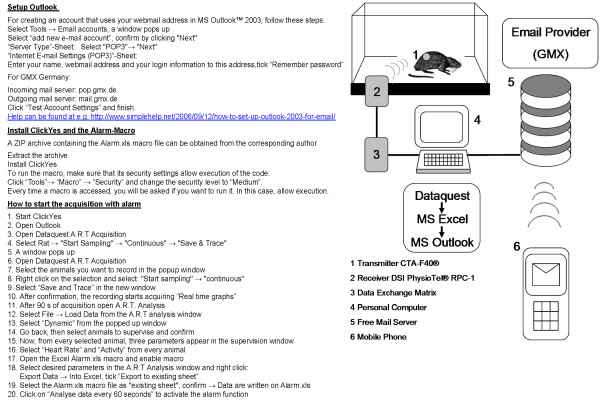
**System configuration and set up**.

The email initiated by the Excel macro is sent via MS Outlook 2003™ (Microsoft, Redmond, WA, USA). In order to circumvent a security query of Outlook, the freeware Express ClickYes 1.2 (http://ContextMagic.com, Vancouver, BC, Canada) was installed. This software automatically confirms the security query with yes, allowing the email to be released. In the GMX account the "instant message-alarm" option was activated and each time an email from the Excel macro was received, the instant message was forwarded. Requirements for the functioning alarm setup are Microsoft Excel, MS Outlook™ with an email account, Express ClickYes 1.2, a Webmail account. For a detailed arrangement instruction please see Figure 1.

With the release of the alarm, the Excel-based vital parameter acquisition from Dataquest stops, displaying the parameter values that triggered the alarm, until it is reset by the experimenter. With the first setup using the heart rate threshold "0", 19 animals participated in the experiment. Four animals died without releasing an alarm. In the 20 weeks that we used the second setup (threshold: heart rate < 150), 37 instant message alarms from 33 rats were triggered via the GMX account. In each case the alarm was sent correctly by means of the heart rate threshold of 150. In 27 animals the alarm was triggered correctly, consequently the respective animals were euthanized in order to reduce suffering and organs harvested in time. One rat died without releasing an alarm and one rat died, releasing an alarm that was not forwarded by GMX. In six animals, the transmitter seemed to be incorrectly placed. Two of those six rats triggered multiple alarms over the entire monitoring period of four days at displayed heart rates below 150 bpm without showing signs of a worsening condition. One false alarm was released by each of four other rats while showing normal, healthy behaviour. To sum up, the alarm systems performance (threshold: heart rate < 150 bpm) is displayed in the confusion matrix (see Table 1).

**Table 1:** Confusion matrix of the alarm (heart rate < 150 bpm)

	Rat Killed	Rat Survived	
Alarm sent to mobile phone	27	6	**33**

Alarm NOT sent to mobile phone	2	57	**59**

	**29**	**63**	**92**

Cave: The six surviving rats that triggered an alarm, released 10 alarms in total!

Positive predictive value (rat dead, alarm sent to mobile phone): 93.1%

Negative predictive value (rat alive, no alarm): 90.5%

Based on the table, a positive predictive value (rat heart rate below 150, alarm sent to mobile phone) of 93.1% and a negative predictive value (rat alive, no alarm) of 90.5% could be obtained. In case of a valid alarm, rats could always be killed and organs harvested in time.

The designed "surveillance and alarm system" seems to provide a reliable and inexpensive extension of the telemetry, to get immediate notification on a mobile phone, if a rat's condition worsens.

The first version with a heart rate condition of zero was of limited utility, since the recorded heart rate never reaches zero due to transmission interferences. After modifying the threshold to heart rate < 150 bpm, the system proved reliable. 150 bpm were chosen because this threshold was too high to be interference related (as seen in the first version with a zero threshold), but low enough to indicate impending death and warrant euthanasia of the respective animal. Organs from 33 rats could be harvested within adequate time and only two animals died without receiving a message on the cell phone. In one case, it remains unclear why the alarm was not released by the Excel macro. The second time no instant message received the experimenter's mobile phone in time due to GMX delaying the forwarding for approximately 12 hours. While our set up worked as required with a correctly triggered alarm and the timely released email to GMX, the forwarding of the instant message was delayed for unknown reasons. To reduce this risk of an email not being forwarded by the webmail provider, MS Outlook™ could be set to send the email notification to two independent webmail accounts simultaneously. Another limitation is the system's use of only the ECG data for surveillance. This limitation can be overcome by either adding the parameter "activity" to the surveillance system or by choosing a different transmitter type that additionally e.g. monitors blood pressure.

Future applications will include investigating the role of body temperature in the early postoperative phase on cerebral outcome after cardiopulmonary bypass with deep hypothermic circulatory arrest. Using this telemetry arrangement will allow us to closely monitor core temperature and keep it in defined ranges according to study design that in turn will determine the macro and alarm settings. The macro as core tool can easily be modified and adapted to a various number of parameters such as body temperature or activity or any combination of it, solely depending on the transmitter being used. Adaption to another webmail provider can also be easily facilitated. The only requirement is a webmail provider capable of releasing instant message alarms to mobile phones.

Establishing this surveillance system helped to avoid uncontrolled death and facilitated timely organ harvesting. Additionally we were able to markedly reduce the drop out rate of experimental animals, and therefore the total number of animals used in our study. With many different transmitters available that record electroencephalograms, blood pressure or any combination of parameters (see DSI website for detailed information), this system can prove a helpful tool to relieve stress and reduce animal numbers in a wide variety of study designs.

## Availability and requirements

Project name: A mobile phone based alarm system for Dataquest^® ^A.R.T 4.0 supervised experiments

Project home page: None

Operating system(s): The alarm Macro is based on Microsoft Windows XP Professional Version 2002 Service Pack 3

Programming language: Microsoft Visual Basic 6.5.

Other requirements: ClickYes1.2 by ContextMagic.com has to be installed. Click yes 1.2 is compatible with Outlook 2000 (SP1+SR1, SP2, SP3), Outlook 2002 (SP1, SP2 and SP3), Outlook 2003 (SP1). An email address at a webmail account, e.g. GMX that supports instant notification via instant message.

License: none

Any restrictions to use by non-academics: none

## Availability of supporting data

The visual-basic macro is available as Additional file 1 with the manuscript. For further questions, please contact the authors.

## Competing interests

### Financial competing interests

• In the past five years have you received reimbursements, fees, funding, or salary from an organization that may in any way gain or lose financially from the publication of this manuscript, either now or in the future? Is such an organization financing this manuscript (including the article-processing charge)?

'The authors declare that they have no competing interests'

• Do you hold any stocks or shares in an organization that may in any way gain or lose financially from the publication of this manuscript, either now or in the future?

'The authors declare that they have no competing interests'

•Do you hold or are you currently applying for any patents relating to the content of the manuscript? Have you received reimbursements, fees, funding, or salary from an organization that holds or has applied for patents relating to the content of the manuscript?

'The authors declare that they have no competing interests'

• Do you have any other financial competing interests?

'The authors declare that they have no competing interests'

### Non-financial competing interests

Are there any non-financial competing interests (political, personal, religious, ideological, academic, intellectual, commercial or any other) to declare in relation to this manuscript?

'The authors declare that they have no competing interests'

## Authors' contributions

KK participated in the study design, carried out the experiments, was involved in the data analysis and helped to draft the manuscript. MK designed and carried out the system setup, was involved in the data analysis, performed the statistical analysis and helped to draft the manuscript. AO designed the macro and participated in the system setup and data analysis. FH carried out the system setup and was involved in the data analysis. EFK participated in the study design and helped to draft the manuscript. BJ participated in the study design, was involved in the data analysis and helped to draft the manuscript. All authors read and approved the final manuscript.

## Supplementary Material

Additional file 1 **The file contains the visual basic macro for data acquisition from Dataquest and messaging routine**. Click here for file
